# Investigation of a monoclonal antibody against enterotoxigenic *Escherichia coli*, expressed as secretory IgA1 and IgA2 in plants

**DOI:** 10.1080/19490976.2020.1859813

**Published:** 2021-01-13

**Authors:** Audrey Y-H Teh, Lisa Cavacini, Yue Hu, Ozan S. Kumru, Jian Xiong, David T. Bolick, Sangeeta B. Joshi, Clemens Grünwald-Gruber, Friedrich Altmann, Mark Klempner, Richard L. Guerrant, David B. Volkin, Yang Wang, Julian K-C. Ma

**Affiliations:** a Molecular Immunology Unit, Institute for Infection and Immunity, St. George’s University of London, London, UK; b MassBiologics of the University of Massachusetts Medical School, Boston, MA, USA; c Vaccine Analytics and Formulation Center, Department of Pharmaceutical Chemistry, University of Kansas, Lawrence, KS, USA; d Division of Infectious Disease and International Health, University of Virginia School of Medicine, Charlottesville, VA, USA; e Department for Chemistry, Division of Biochemistry, Universität Für Bodenkultur Wien, Vienna, Austria

**Keywords:** Enterotoxigenic Escherichia coli, monoclonal antibody, secretory IgA, passive immunization, immunotherapy, Nicotiana benthamiana

## Abstract

Passive immunization with antibodies is a promising approach against enterotoxigenic *Escherichia coli* diarrhea, a prevalent disease in LMICs. The objective of this study was to investigate expression of a monoclonal anti-ETEC CfaE secretory IgA antibody in *N. benthamiana* plants, with a view to facilitating access to ETEC passive immunotherapy. SIgA1 and SIgA2 forms of mAb 68–81 were produced by co-expressing the light and engineered heavy chains with J chain and secretory component in *N. benthamiana*. Antibody expression and assembly were compared with CHO-derived antibodies by SDS-PAGE, western blotting, size-exclusion chromatography and LC-MS peptide mapping. N-linked glycosylation was assessed by rapid fluorescence/mass spectrometry and LC-ESI-MS. Susceptibility to gastric digestion was assessed in an *in vitro* model. Antibody function was compared for antigen binding, a Caco-2 cell-based ETEC adhesion assay, an ETEC hemagglutination inhibition assay and a murine *in vivo* challenge study. SIgA1 assembly appeared superior to SIgA2 in plants. Both sub-classes exhibited resistance to degradation by simulated gastric fluid, comparable to CHO-produced 68–61 SIgA1. The plant expressed SIgAs had more homogeneous N-glycosylation than CHO-derived SIgAs, but no alteration of *in vitro* functional activity was observed, including antibodies expressed in a plant line engineered for mammalian-like N glycosylation. The plant-derived SIgA2 mAb demonstrated protection against diarrhea in a murine infection model. Although antibody yield and purification need to be optimized, anti-ETEC SIgA antibodies produced in a low-cost plant platform are functionally equivalent to CHO antibodies, and provide promise for passive immunotherapy in LMICs.

## Introduction

Enterotoxigenic *Escherichia coli* (ETEC) causes severe diarrhea^1^, commonly in the first 2 y of life^2^. With estimates of several hundred million cases of diarrhea each year, mostly in low- and middle-income countries (LMICs), ^3^ ETEC is a leading cause of death among young children, with an estimated mortality of 300–500,000 in children under 5 y.^4^ ETEC is also estimated to cause approximately 10 million episodes of travelers’ diarrhea each year.^5^ A systematic review indicated that ETEC was detectable in 30–40% of travelers with diarrhea, particularly in endemic regions.^6^

ETEC is transmitted by the oro-fecal route through contaminated water or food. The primary control strategy is prevention of transmission through building sanitation infrastructure and basic food and water hygiene measures. In adults, ETEC diarrhea may be helped by a short course of antibiotics, but the development of antibiotic resistance is increasingly reported.^7,8^

There is currently no commercial vaccine against ETEC. Vaccine development is challenging, due to antigenic diversity, including two enterotoxins ^9^ and over 25 filamentous bacterial surface structures known as colonization factors and coli surface antigens.^10^ A killed whole cell vaccine (Dukoral®), primarily designed and licensed to prevent cholera, contains a recombinant B subunit of the cholera toxin that is antigenically similar to the heat labile toxin of ETEC and has been recommended by some, ^11^ but a Cochrane review of twenty four randomized controlled trials did not provide sufficient evidence to support this intervention.^12^

Promisingly, protective immunity to ETEC has been demonstrated after both natural and experimental infection. In endemic areas, ETEC infection declines after 3 y of age suggesting acquisition of immunity, ^13^ and in human studies, subjects who recovered from ETEC diarrhea were protected against new infections with ETEC.^14^ Vaccine strategies have focused on eliciting anti-toxin antibodies and anti-colonization factor immunity, as antibodies against both targets can contribute to protection.^15,16^ As ETEC infections are confined to the mucosal surfaces of the gut, it is generally considered that secretory IgA antibodies are likely to play an important role in immune protection.^17^ In a piglet ETEC model, monoclonal IgA mixed into food was reported to prevent infection.^18^

CfaE is the minor subunit of CFA/I, one of the most important colonization factors expressed by pathogenic ETEC strains ^19^ and is responsible for adhesion to host intestinal epithelium. CfaE was previously shown to elicit protective antibodies that provided passive immunity against infection in animals and humans.^20,21^ Recently, the development of a panel of 360 human monoclonal antibodies (mAb) against CfaE was reported.^22^ Three of these that were class-switched and expressed as SIgAs were further tested in a murine ETEC colonization model, and demonstrated a 2–4 log decrease in colony formation in comparison to animals treated with irrelevant SIgA controls.

With the aim of improving access to new SIgA products, in this study, we explore the feasibility of using anti-CfaE IgAs produced in plants, as oral immunotherapy for ETEC. Plants are increasingly attracting attention as a potential manufacturing platform for biologics like monoclonal antibodies and vaccines, ^23^ particularly those that are primarily needed in developing parts of the world. They offer important potential advantages, including low cost, massive scalability and rapid manufacture, as well as an opportunity to transfer technology to establish new manufacturing capacity in less developed regions.^24^ Several plant-made antibodies have already entered clinical trials.^25,26^ Plants were also the first heterologous expression system described for recombinant secretory IgA antibodies ^27^ and an early human clinical trial using an orally delivered SIgA produced in transgenic tobacco has been reported.^28^

We selected the most potent anti-CfaE SIgA, 68–61 and manufactured this as recombinant SIgA1 and SIgA2 in *Nicotiana benthamiana*. The use of glycoengineered plant expression hosts has become standard in recent years to avoid glycoforms that are not usually found in humans, ^29^ so the use of such engineered lines was investigated here. The objective was to assess plant-produced SIgAs, comparing to SIgAs produced in CHO cells in respect to key preliminary evaluations of structural analysis, functional analysis of antigen binding and functional properties of SIgA.

## Results

### Plant secretory IgA assembly and identity

mAb 68–61 alpha1 or alpha2 heavy chain with kappa light chain was expressed with human J chain and secretory component by co-infiltration with the four relevant recombinant agrobacterium strains in *N. benthamiana*. Two *N. benthamiana* lines were used, one with unaltered (WT) plant glycosylation and another (ΔXF) in which glycosylation is altered by deletion of xylosyl – and fucosyl-transferases.

**Figure 1.**
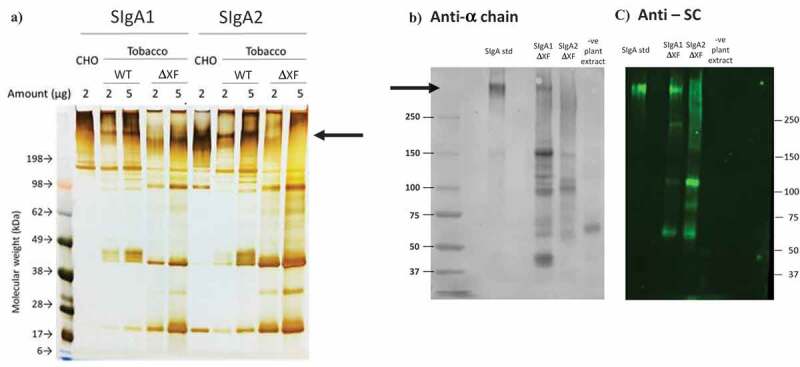
Comparison of human ETEC 68–61 SIgA1 and SIgA2 prepared in CHO cells or plants. Non-reduced samples were separated by SDS-PAGE. (a) Silver stained polyacrylamide gel separating 2 or 5 µg of total protein per lane; (b) Western blot of plant produced 68–61 SIgA1 and SIgA2. Detection with HRPO-labeled sheep anti-human alpha chain serum and DAB; (c) Western blot of plant antibodies and detection with mouse anti-secretory component serum, and fluorescein-labeled anti-mouse IgG serum. SIgA1 or SIgA2 were produced in CHO cells, wild-type (WT) or ΔXF tobacco as indicated. SIgA std is a polyclonal SIgA preparation from human colostrum. Arrows depict putative SIgA bands

After 5 d, total plant leaf extracts were prepared and recombinant antibody was affinity purified. The samples were analyzed by non-reducing SDS-PAGE with silver staining and a representative result is shown in Figure 1a. Purified SIgA1 and SIgA2 antibodies prepared in CHO cell culture are shown for comparison. mAb 68–61 SIgA1 expressed in WT plants contains a prominent band of the expected molecular size (arrow). Smaller prominent bands in the Mr90-200 K range are also detected, possibly representing assembly intermediates, which are also present in the CHO preparation. In the plant antibody samples, there were also a number of lower molecular weight bands (<Mr50K), which may represent degradation products. The SIgA2 sample resolved similarly to SIgA1. When expressed in the ΔXF plant line the major SIgA1 antibody bands appeared to have faster mobility, possibly reflecting lower molecular weight. Detection of SIgA2 expressed from the ΔXF plant line was much reduced. The overall size and aggregation profile under non-denaturing conditions were evaluated by SEC.^30^ Each SIgA sample displayed a heterogeneous mixture of molecular weight species including protein at the expected molecular weight for SIgA as well as various lower molecular weight species. In addition, higher molecular weight material was observed in all samples indicating the presence of some polymeric or aggregated material (Suppl Figure S1).

The identity of the protein bands in SDS-PAGE was confirmed by western blotting using specific antisera against the alpha heavy chain (panel B) or secretory component (panel C). Here, a commercial SIgA preparation purified from human colostrum (Sigma) was used as a positive control (SIgA std). The high molecular weight band assumed to represent SIgA1 was confirmed to include alpha chain and secretory component (SIgA1 ΔXF). No distinct band was observed at high molecular weight for plant SIgA2 (SIgA2 ΔXF). The probability that the lower molecular weight bands observed in SDS-PAGE are assembly intermediates or degradation products was supported by their detection in the western blot using both anti-alpha chain and anti-SC. An extract from an untransformed plant served as a negative control and no cross-reactive proteins were identified (-ve plant extract).

LC-MS peptide mapping confirmed the presence of each of the polypeptide chains in the purified SIgA1 and SIgA2 samples. The results indicated 88–99% coverage of the light chain in SIgA1 and SIgA2 from both WT and ΔXF plants, 60–72% coverage of the respective alpha heavy chains, 48–88% coverage of the J chain and 41–61% coverage of the secretory component (data not shown).

### Binding to cognate antigen

**Figure 2.**
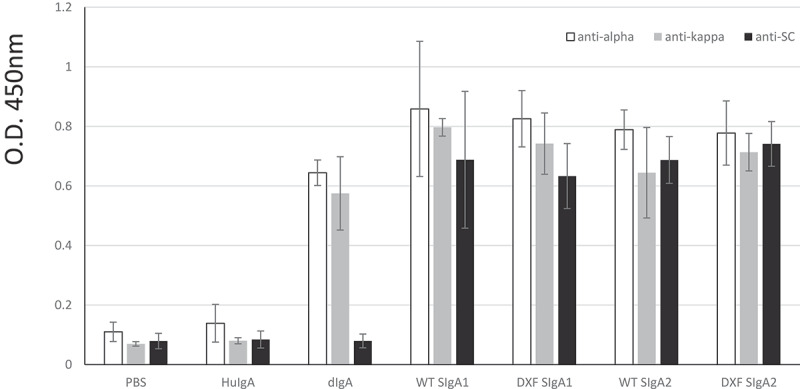
Binding of anti-ETEC 68–61 SIgAs to MBP-CfaE antigen. Individual components of the SIgAs were detected by either anti-alpha chain, anti-kappa chain, or anti-Secretory Component antiserum. 1ug/mL anti-ETEC dimeric IgA (Mass. Biologics) was used as positive control. PBS and nonspecific human colostral SIgA (HuIgA) were used as negative controls. Plant extracts were loaded at 4-fold dilutions. Results are shown as mean+sd of triplicate assays

Recognition of specific ETEC CfaE antigen was determined by ELISA (Figure 2). ELISA plates were coated with the MBP-CfaE antigen and after blocking, incubation was with the four types of plant antibody (SIgA1 and SIgA2 from WT or ΔXF plants). Detection of binding was with anti-alpha, anti-kappa or anti-secretory component antisera. Here, the positive control was CHO-derived dimeric mAb 68–61 IgA (dIgA), which gave a positive signal when tested with anti-alpha and anti-kappa chain antisera, but not anti-SC antiserum as expected. The negative controls were PBS and nonspecific human colostral SIgA (HuIgA) which demonstrated no binding to ETEC antigen. Antigen binding by all the plant SIgA1 and SIgA2 samples was demonstrated using all antisera. Inconsistent binding by anti-J chain antiserum was detected (not shown), which has been reported previously by us and others.^27,31^

The yield of SIgA antibodies was determined by capture ELISA, using a standard curve derived from purified human colostral SIgA (not shown). Taking an average from three batches for each antibody, the yields of purified antibody/kg of fresh leaf tissue were: SIgA1 (WT *N. benthamiana) –* 7.1 mg; SIgA1 (ΔXF *N. benthamiana) –* 8.7 mg; SIgA2 (WT *N. benthamiana) –* 1.1 mg; and SIgA2 (ΔXF *N. benthamiana) –* 2.6 mg.

### Glycoanalysis of CHO and plant-derived SIgA1 and SIgA2

A broad analysis of N-glycosylation in the CHO and plant-derived SIgA1 and SIgA2 mAbs was performed using a rapid fluorescent/Mass spectrometry approach (Table 1). The results demonstrated greater heterogeneity in the N-linked glycoform species from CHO-derived antibodies than those from tobacco, particularly those of the complex glycan types. Glycans associated with SIgA1 and SIgA2 produced in the same host system were similar. As expected, some glycoforms were only associated with CHO manufacture and others with plant expression. For the latter, as expected, the XA1, XM3 and XA2 N-linked glycoform species were identified in WT *N. benthamiana* produced antibodies, but they were virtually absent in ΔXF *N. benthamiana* produced antibodies.

**Table 1.** Summary of N-glycosylation identification of 68–61 SIgA1 and SIgA2 produced in CHO, *N. benthamiana* and ΔXF *N. benthamiana* as measured by LC-MS glycan analysis. Check marks indicate observed N-glycosylation species from each individual SIgA sample. N-glycans are given according to the Consortium for Functional Glycomics notation; the Oxford glycan nomenclature was used for the abbreviations

A more comprehensive glycoanalysis was performed of the WT and ΔXF *N. benthamiana* produced SIgA1 and SIgA2 antibodies, using LC-ESI-MS. In this analysis, N-glycosylation sites on the alpha chains, J chain and secretory component were assessed quantitatively and individually as well as a potential O-glycosylation site on the alpha1 chain. Alpha1 heavy chains have two potential N-glycosylation sites and alpha2 heavy chains have four; J chain has one potential N-glycosylation site; and SC has five. The results were consistent with the findings from the Rapi-Fluor preliminary analysis. In addition, the analysis demonstrated that in the plant-produced SIgA1 antibodies, all potential N-glycosylation sites were occupied on the heavy and J chains, but no glycans could be detected associated with glycosites 1, 3 and 4 in secretory component (Suppl. Figure S2). Glycosite 2 on the alpha1 chain was ~30% non-glycosylated, suggesting reduced accessibility of this site, compared with glycosite 1. The major glycoforms are shown, with a preponderance of xylosylated (XA1) and xylosylated and fucosylated (FXA1, FXA2) glycoforms on the alpha chain and secretory component. In the ΔXF *N. benthamiana* produced antibodies, the results support the elimination of xylosylation and a significant knock-down of fucosylation.

The N-glycan profile of plant-produced SIgA2 was very similar to SIgA1 (Suppl. Figure S3). Glycosylation was not detected on the alpha2 chain at glycosites 1 and 3. Glycosites 2 and 4 were modified almost identically to alpha1 chain glycosites 1 and 3, respectively. Interestingly, the glyco-engineering observed in the ΔXF plant host was highly consistent, resulting in virtually the same glycan changes in alpha1 and alpha2 chains. J chain was glycosylated very similarly in SIgA1 and SIgA2 with the majority of glycoforms of the high mannose type. For SC, glycosylation at glycosites 1, 3 and 4 was not detected. Site 2 was glycosylated but only at the limit of detection in our system, so detailed information is not provided. Glycosite 5 was the only site where clear data was obtained, and like J chain there was no difference between SIgA1 and SIgA2. For both J chain and SC, the effect of glyco-engineering in ΔXF plants was identical.

The proline residues of the O-glycosylation site of SIgA1 were partially oxidized to hydroxyprolines, which themselves were partially occupied by arabinose chains of varying length (Suppl. Figure S4). A relatively complex profile of peptide variants was present for the hinge region peptide of SIgA1 with no obvious difference between the plant wild type and ΔXF *N. benthamiana* produced antibodies.

### Susceptibility of SIgAs to degradation under in vitro gastric digestion conditions

**Figure 3.**
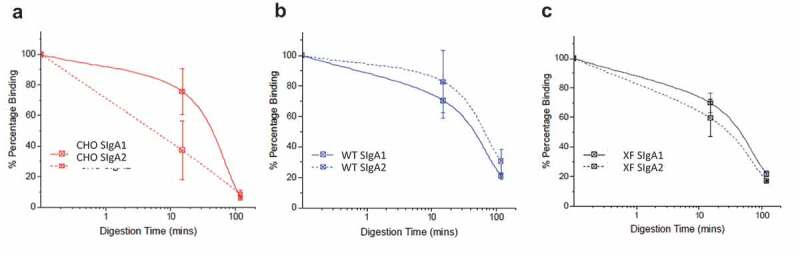
*In vitro* gastric digestion model showing stability profiles of SIgA1 and SIgA2 produced in CHO cells and *Nicotiana benthamiana*. Comparison of cfaE-antigen binding for anti-cfaE mAbs (SIgA1 and SIgA2) after incubation in an *in vitro* gastric digestion model as measured by ELISA. The SIgA mAbs were produced in (a) CHO cells, (b) WT, and (c) ΔXF *N. benthamiana*. The relative percent of cfaE antigen binding remaining for each mAb was normalized to time zero binding. Each data point is displayed as the mean ± the data range; n = 2

CHO and plant-produced SIgA1 and SIgA2 mAbs were subjected to pepsin digestion at pH 3.5 in modified simulated gastric fluid. Antibody degradation was measured by a cfaE antigen binding ELISA (Figure 3). For the CHO-produced mAbs, SIgA1 appeared to retain more antigen binding ability after approximately 15 minutes of digestion with pepsin, compared to SIgA2, but there were no notable differences after 100 minutes. For the WT and ΔXF *N. benthamiana* produced antibodies, there were no notable differences in the digestion profiles of SIgA1 and SIgA2, which were both similar to the CHO SIgA1.

### In vitro functional efficacy

The functional activity of different mAb 68–61 preparations was compared using an ETEC adhesion assay with Caco-2 cells (Table 2). The minimum dose for 60% inhibition of cell adhesion was in the sub-microgram/ml levels for all samples. There was no notable difference between SIgA1 and SIgA2 samples and the plant antibodies performed as well as the CHO-produced antibodies.

**Table 2.** In vitro activity against ETEC of 68–61 SIgA1 and SIgA2 produced in CHO, *N. benthamiana* and ΔXF *N. benthamiana*. The minimum dose for 60% inhibition of ETEC adhesion to Caco-2 cells is shown on the left side; the minimum dose for 100% inhibition of ETEC hemagglutination is shown on the right. Results are the mean of a minimum of three experiments

A mannose-resistant hemagglutination assay of human erythrocytes was also performed with similar results. The minimum dose for 100% inhibition of ETEC induced hemagglutination was also in the sub-microgram/ml range and there were no differences between any of the SIgA antibody samples.

In both assays, there was no activity for irrelevant antibody controls.

### In vivo protection against ETEC challenge

**Figure 4.**
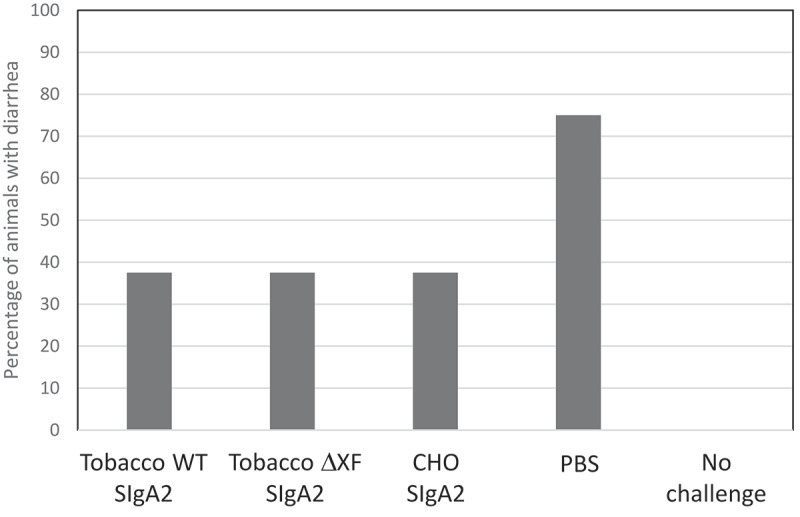
*In vivo* protection against ETEC challenge. Groups of 8 mice were inoculated with ~1x10^9^ *Escherichia coli* (H10407) mixed with 68–61 SIgA2 produced in *N. benthamiana* WT, ΔXF SIgA2 or CHO cells, or PBS only. In addition, a group of 12 mice were untreated and not infected. The percentage of mice developing diarrhea within 7 d is shown. Diarrhea was defined as unformed or watery stools occurring on any day of daily observations in each mouse

The protective efficacy of CHO-produced and plant-produced SIgA2 was next tested in a murine infection model. 10^9^ colony-forming units of ETEC strain H10407 were incubated with antibodies or PBS for 1 hour, before being administered in 100 μl volume by oral gavage to groups of eight C57BL/6 mice. The readout was onset of diarrhea by 7 d post-infection and the results are shown in Figure 4. In the three groups where CHO-produced or plant-produced antibodies 68–61 SIgA mAbs were used, only 37.5% of animals developed diarrhea and there was no difference between groups. In the control group, where no mAb was added, a significantly higher number, 75% of the mice developed diarrhea (p < .0001; two-tailed binomial test). None of the 12 non-challenged mice developed diarrhea.

## Discussion

Passive immunization with anti-ETEC antibodies has been demonstrated in animal models^32^ and human volunteers.^21^ In the latter Phase I study, antibodies directed against the CFA/I minor pilin subunit (CfaE) protected against ETEC challenge, demonstrating that fimbrial tip adhesins are protective antigens. Importantly, the hyperimmune bovine IgG antibodies were delivered by the oral route, three times/day for one week, starting 2 d prior to ETEC challenge, so these findings opened the way for development of improved and affordable orally delivered products.

For oral delivery, IgG antibodies are not optimal owing to their susceptibility to degradation at mucosal surfaces.^28^ Secretory (S)IgA antibodies are preferred. They are the major naturally occurring form of antibodies in mucosal secretions with specific adaptations for the mucosal environment. In humans, IgA exists as two subclasses, IgA1 and IgA2 both of which can assemble further into SIgA1 and SIgA2, respectively.^33^ Both are found in the gastrointestinal tract.^34^

These two IgA subclasses have arisen through gene duplication, and hence share considerable sequence similarity. The major structural difference is in the hinge region, where IgA1 features an extended hinge comprising two copies of an 8 amino acid sequence, decorated with up to 6, O-linked oligosaccharides.^35^ The longer IgA1 hinge may be an adaptation to enable higher avidity engagement with widely spaced antigens,^36^ but it also increases susceptibility to proteolytic attack.^37^

The extended hinge of IgA1 may not be the only important consideration in selecting antibody format. Other significant differences exist between IgA1 and IgA2, such as the extent of non-covalent binding of SC to IgA2^38^ which may explain the difficulty that has consistently been experienced in expressing and purifying SIgA2. The different binding of SC to IgA1 and IgA2 was also shown to affect proteolytic degradation.

In this study, we compared SIgA1 and SIgA2 versions of the same anti-ETEC antibody produced in CHO and *N. benthamiana* platforms. In *N. benthamiana*, the yield (1–9 mg/kg fresh leaf weight) was consistent with that of a human SIgA reported previously.^31^ IgG mAbs however can be expressed at yields of 400 mg/kg fresh leaf weight,^39^ so further work is required to optimize expression of these anti-ETEC SIgAs in *N. benthamiana.*

The results suggest that the plant-produced SIgA antibodies are similar to antibodies expressed in CHO cells, with no differences in antigen recognition and binding, as expected. However, some differences were noted and opportunities for improvements in product quality were identified. For example, increased low molecular weight impurities in the plant samples need to be addressed with optimized purification procedures to better remove trace proteases.^40^ In plants, SIgA1 assembly appeared superior to that of SIgA2, particularly in the ΔXF plant line, although ELISA assays indicated the presence of fully assembled SIgA in both SIgA1 and SIgA2 samples. The apparent difference between SIgA1 and SIgA2 assembly has been reported previously,^38^ and several factors could be involved. Differences in SC interactions with α1 and α2 heavy chains have been discussed above. There are also amino acid sequence differences between α1 and α2 chains throughout the constant region domains. Indeed, comparing the heavy chain sequences used here, there were 7 amino acid differences in the Cα1 domain and 10 amino acid differences in the Cα2 domain. The Cα3 was identical, but the possibility that amino acid changes could result in cryptic targeting sequences affecting protein assembly, accumulation or stability needs to be addressed further.

There was little functional difference between SIgA1 and SIgA2 either in the Caco2 cell adhesion assay or the hemagglutination assay. The SIgA2 was selected for the mouse challenge study because it is potentially a better clinical candidate with respect to stability and resistance to degradation in the gut environment. It demonstrated equivalent protection to that provided by CHO-produced SIgA2.

We also produced secretory mAbs in a glycoengineered *N. benthamiana* line,^29^ the rationale for which was the avoidance of β1-2 xylose and α1-3 fucose, which are non-human glycoforms. N-glycan analysis of the antibodies produced in ΔXF plants demonstrated a consistent elimination of xylosylation and virtually complete elimination of fucosylation. Importantly, neither of these two N-glycan modifications had any significant effect on antibody expression, biochemical analysis or protective efficacy.

Differences in glycosylation might also affect protein assembly efficiency and/or susceptibility to degradation. The overall glycan composition was identified with the unexpected finding that sialylated complex glycans were not observed in the CHO 68–61 SIgAs. Other CHO-produced SIgAs have been reported to be sialylated^30^ and sialylation can affect serum IgA mediated effector functions,^41^ so this result merits further study. Sialylation is not found in plants, although the pathway can be engineered.^42^ A more detailed site-specific analysis of the plant antibodies was performed. α1 heavy chains are commonly glycosylated at two sites whilst α2 heavy chains are usually glycosylated at four. In this study, α1 heavy chain N-glycosylation sites were fully occupied, whereas only two α2 heavy chain N-glycosylation sites (sites 2 and 4) appeared to be utilized. Furthermore, in contrast to our previous report with a different SIgA where 6 of the 7 potential N-glycosylation sites on SC were occupied, only 2 glycosites were found on SC in mAb 68–61 SIgA1 and SIgA2. An important role of SC is to protect dimeric IgA from proteolytic degradation^43^ so resolving this discrepancy might also be a priority for future work.

α1 heavy chains also contain O-linked glycans. Three to six mucin-type O-glycans are commonly attached to the nine potential O-glycosylation sites in the hinge region of human IgA1.^44^ Our results confirmed the presence of typical plant-like O-glycosylation on all IgA1 samples, consisting of hydroxylated proline residues with attached arabinose residues. Thus, it is also possible that assembly and stability of SIgAs is affected by O-linked sugars.

Conclusive evidence supporting the choice of either SIgA1 or SIgA2 for mucosal passive immunization remains to be determined, but the ultimate goal of preventing ETEC disease in newly born children by passive oral immunization with specific monoclonal secretory antibodies may now be achievable. CHO cell-based manufacture of SIgAs is feasible, but it is unlikely that the CHO platform could ever be economically viable for an orally delivered product, particularly one targeted at neonates in LMICs.^45^ Other groups that might benefit from a short-term use of orally delivered SIgA are travelers or military personnel.

This study indicates that protective anti-ETEC SIgA1 and SIgA2 antibodies can both be produced by *N. benthamiana*, and whilst further work is needed to consider best antigenic targets, the possibility of combining mAbs, to optimize alpha chain constant region sequences, maximize yields and establish more efficient extraction and purification processes, this would be a requirement for any expression system. A long-term aspiration, requiring more regulatory development, would be to express anti-ETEC secretory antibodies in edible plants, allowing direct administration by the oral route, as has been demonstrated by vaccine delivery using corn and potatoes for diarrheal and other diseases.^46,47^ This would simplify extraction and downstream processing, steps that are generally regarded as the major contributors to cost of goods.^48,49^

## Materials and methods

### Anti-ETEC mAb 68-61 gene constructs

The heavy and light variable region genes of mAb 68–61 were codon optimized for Nicotiana, synthesized (Geneart, USA) and cloned into pDONOR-based plasmids between a human Ig heavy chain leader sequence and human alpha 1 or alpha 2 constant region, or a human light chain leader sequence and human kappa chain constant region. Full length heavy and light chain genes were sub-cloned into MIDAS entry vectors, containing the CaMV 35s double promoter before being combined into the pTRAK.6 destination vector. A pTRAK.6 vector for both IgA1 and IgA2 was prepared, and introduced into *Agrobacterium tumefaciens* strain GV3101 PMP90/RK by electroporation.

Genes encoding human secretory component (SC) and J chain were synthesized and cloned into separate pEAQ-HT vectors.^2^ The pEAQ-HT vectors were electroporated into *Agrobacterium tumefaciens* strain LBA4404.

### Vacuum infiltration with N. benthamiana

Agrobacteria containing appropriate constructs were grown overnight at 28°C in Lysogeny-Broth (LB), 100 µg/mL rifampicin, 50 μg/mL carbenicilllin and 5 µg/mL kanamycin for MIDAS constructs and 100 µg/mL rifampicin and 50 µg/mL kanamycin for pEAQ-HT constructs. After centrifugation, the bacterial pellet was resuspended to OD_600_ with Infection Solution (IS; 0.01 mM MES and 0.01 mM MgCl_2_). The agrobacteria was introduced at a 2:4:1 (alpha/kappa:J:SC) ratio into 6–8 week wild-type (WT) or glycoengineered ΔXF *Nicotiana benthamiana* ^50^ by vacuum infiltration.^51^ Plants were further grown in a controlled environment room at 25°C with 16/8 hour light/dark cycle.

### Protein purification

Vacuum infiltrated leaves were harvested after 6 d. Leaf extracts were prepared with 3 volumes of PBS (pH8.0) with 0.1% Tween 20. Clarified crude extracts were purified using Capto-L^TM^ (GE Healthcare, USA) column. After washing, the protein was eluted with 0.1 M glycine-HCl (pH2.7) and neutralized with 1 M Tris-HCl (pH9.0). The antibody was dialyzed against PBS 0.01% Tween 20 (Slide-A-Lyzer 100kDa; Thermo Scientific, USA) and concentrated using Amicon Ultra-15 (molecular cutoff 100kDa; Milipore, Ireland). The concentrations of purified antibodies were determined by ELISA.

### PAGE gel and Western blot

Purified SIgAs were resolved on a NuPage 3–8% Tris Acetate gel (Life Technologies, UK) and stained with InstantBlue (Expedeon, UK). For the silver stained gel, samples were separated on 10% NuPAGE Bis-Tris gels (Life Technologies) and visualized using a silver stain kit (Thermo-Fisher). For Western blots, resolved gels were blotted onto nitrocelullose membrane (GE Healthcare, USA) and detected using 1:2500 goat anti-alpha chain-HRP (Sigma, USA), 1:2500 sheep anti-kappa chain-HRP (The Binding Site, UK), or 1:1000 mouse anti-SC (Sigma, USA) antisera, followed by 1:2000 IRDye® 800CW goat anti-mouse IgG (LI-COR Biosciences, USA) or 1:1000 rabbit anti-J chain (Sigma, USA) antisera followed by 1:2000 anti-rabbit-HRP antiserum (The Binding Site, UK). Human colostrum SIgA (Sigma, USA) was used as positive control. Detection was performed using the ECL Prime system (Pierce, USA) and visualized using G:Box F3 (Syngene, UK).

### Immunosorbent assays

For antibody characterization, 2 ug/mL MBP-CfaE was coated on ELISA plates overnight at 4°C. After blocking with 1% BSA in PBS/0.1% Tween 20, purified antibody samples were incubated with appropriate controls. Bound antibodies were detected with 1:1000 goat anti-alpha chain-HRP (Sigma, USA), 1:1000 sheep anti-kappa chain-HRP (The Binding Site, UK), 1:1000 mouse anti-SC (Sigma, USA) antisera, followed by 1:1000 goat anti-mouse IgG-HRP (Sigma, USA) or 1:1000 rabbit anti-J chain (Sigma, USA) antisera, followed by 1:1000 anti-rabbit-HRP antiserum (The Binding Site, UK) followed by visualization with TMB substrate.

For antibody quantification, a similar assay was performed. ELISA plate coating was with 1:200 of mouse anti-SC antiserum (Sigma, USA) and detection was with 1:1000 sheep anti-kappa-HRP (The Binding Site, UK). Purified SIgA from human colostrum (Sigma, USA) was used to derive a standard curve.

### LC-MS Peptide Mapping

LC-MS peptide mapping was performed as described elsewhere.^30^ Briefly, 50 μL of 1 mg/mL mAb samples were reduced and denatured with 3 μL of 0.5 M DTT and 10 μL of 6 M guanidine hydrochloride, then alkylated with IAM prior to overnight trypsinization (~1:25 enzyme:mAb ratio) at 37°C. After trypsin inactivation, the samples were treated with PNGase F (New England BioLabs, Ipswich, MA). Prior to LC-MS, 0.05% (v/v) trifluoroacetic acid was added, and samples were centrifuged for 5 min at 14,000 x g.

The peptides were separated by reversed phase UHPLC (Thermo Scientific) using a C18 column (1.7 μm, 2.1 × 150 mm, Waters Corporation). Mass spectrometry analysis was performed using a LTQ-XL ion trap (Thermo Scientific) and Xcalibur v2.0 software (Thermo Scientific).^30^

### N-Glycan Oligosaccharide analysis

N-Glycan oligosaccharide analysis was performed^30^ using the GlycoWorks RapiFluor-MS N-Glycan Kit (Waters Corporation, Milford, MA). Fluor-MS N-glycan analysis was performed using an Agilent 1260 Infinity II HPLC system equipped with a 1260 FLD detector and an Agilent 6230 electrospray ionization Time-of-Flight mass spectrometer (Agilent, Santa Clara, CA). A HILIC AdvanceBio Glycan Mapping column (120 Å, 2.1 × 150 mm, 2.7 μm), operated at 45°C, was used to separate various N-glycans. Fluorescence was obtained using excitation and emission wavelengths of 265 and 425 nm, respectively. MS was acquired simultaneously from 400 to 2000 m/z at a constant scan rate of one spectrum per second. N-glycans were assigned based on m/z values using a N-glycan database (Water/NIBRT Glycan 3+) and N-glycan quantification was calculated on integration of the fluorescence chromatogram.

Site-specific glycosylation analysis was also performed as described previously.^52^

### Small scale, in-vitro model of gastric digestion and cfaE ELISA

The *in vitro* gastric digestion model to examine mAb stability was performed as described previously, ^30^ using simulated gastric fluid (94 mM NaCl, 13 mM KCl, 0.15 mM CaCl2 with 10 mM citrate-phosphate buffer pH 3.5). The reaction was started with 2000 U/mL pepsin (Sigma, US) and incubation was at 37°C for varying amounts of time. The reactions were neutralized by addition of 0.4 M NaOH and diluted to 1 μg/mL in ELISA blocking buffer (1% BSA in PBS) and stored at −20°C. ELISA was performed as described previously.^22^

### CHO 68-61 SIgA and dIgA antibody production and characterization

68–61 SIgA2 antibody was produced and characterized in CHO cells as previously described.^22^ Antibody was purified by CaptoL resin (GE Life Sciences) followed by size exclusion chromatography (HiLoad 26/600 Superdex 200 pg size exclusion column; GE Life Sciences).

### Mannose-resistant hemagglutination assay of human group A erythrocytes

In a U-bottom 96-well plate (Nunc Thermo Scientific) SIgA antibodies were serially diluted 1:2 in duplicate and an equal volume of H10407 ETEC (ATCC35401) at an OD600nm of 1, was added to each well with 0.1 M D-mannose solution (Sigma, USA). After a 10 minute incubation at room temperature, human erythrocytes type A+ (BioreclamationIVT) were added to the plate at a final concentration of 1.5% (vol/vol) and mixed well. Hemagglutination was measured after 2 hours at 4°C.

### Analytical size exclusion chromatography

SEC was performed as described,^30^ using a Shimadzu Prominence ultra-fast liquid chromatography HPLC system. 10 µL of mAb (10 µg total protein) was injected and separated by a TSKgel G4000SWXL column (8 µm particle size, 7.8 mm ID × 30 cm) with the corresponding guard column operated at ambient temperature (Tosoh Biosciences) using a 30-minute run time. Gel filtration molecular weight standards (Bio-Rad, Hercules, CA) were injected as controls. Data were analyzed using LC-Solutions software (Shimadzu, Kyoto, Japan).

### Caco-2 adhesion assay

ETEC bacteria grown on CFA agar were resuspended to an OD690nm of 0.1. Caco-2 cells were seeded at 1 × 10^5^ cells/mL in 24-well tissue culture plates containing Dulbecco’s modified Eagle’s medium at 37°C in 5% CO_2_. Antibody dilutions and bacteria were combined in a 1:10 ratio and incubated for 1 hour at room temperature with shaking (300rpm), after which 0.2 mL of antibody/bacteria mixture was added to Caco-2 cells. The plates were incubated for 3 hours at 37°C and the cells washed to remove non-adherent ETEC cells. Caco-2 cells were dislodged with 0.25% trypsin, collected via centrifugation and resuspended in PBS. Dilutions were plated on CFA agar plates and colonies counted the next day. IC50 was defined as concentration of antibody needed to inhibit 50% of ETEC adhesion to the Caco-2 cells, compared to an irrelevant isotype antibody.

### Murine model testing in vivo

#### Animal husbandry

The murine study according to recommendations in the Guide for the Care and Use of Laboratory Animals of the National Institutes of Health. The protocol was approved by the Committee on the Ethics of Animal Experiments of the University of Virginia (Protocol Number: 3315) in accordance with the Institutional Animal Care and Use Committee policies of the University of Virginia. All efforts were made to minimize suffering. Mice were male, 28 d old, C57BL/6 strain (Jackson Laboratories, ME), and maintained on a standard rodent diet (Harlan).

Following a short acclimation period, the mice were given gentamicin (35 mg/L), vancomycin (45 mg/L), metronidazole (215 mg/L), and colistin (850 U/ml) in drinking water for 3 d.^53^ The mice were then given untreated water for 1 d, followed by a single oral challenge by gavage of ETEC.

#### Antibody treatment and ETEC infection

ETEC (H10407) cultures were grown from glycerol stocks in DMEM at 37°C in a shaking incubator.^54^ Infected mice received an inoculum ~1x10^9^ ETEC in 100 µL (90 µL antibody – approx. concentration 3 mg/ml, or PBS + 10 µL DMEM); controls received 100 µL of PBS alone.

Five groups (n = 8) were: Uninfected, ETEC+PBS, ETEC+CHO SIgA, ETEC+WT SIgA2, and ETEC+ΔXF SIgA2. The mice were euthanized on day 7 after infection.

## Supplementary Material

Supplemental Material

Supplemental Material
