# Supplementary material for: Investigation of a monoclonal antibody against enterotoxigenic Escherichia coli, expressed as secretory IgA1 and IgA2 in plants
Source: Gut Microbes. 2021 Jan 13;13(1):1859813. doi: 10.1080/19490976.2020.1859813 (PMC7833773; doi:10.1080/19490976.2020.1859813)
Supplement: Supplemental Material [file KGMI_A_1859813_SM5320.pptx]

## Slide 1
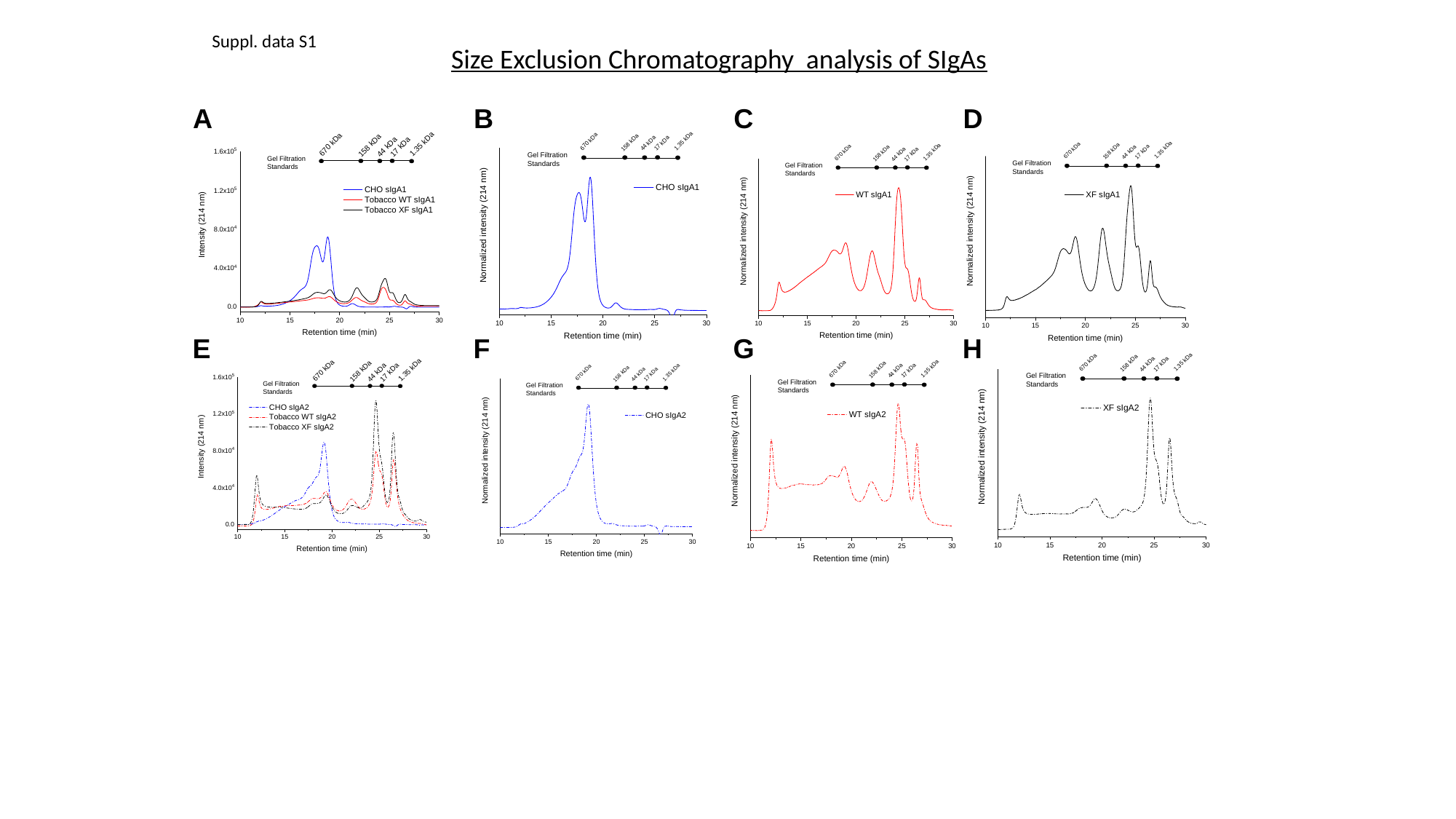

Suppl. data S1
Size Exclusion Chromatography analysis of SIgAs
A
B
C
D
### Chart
| Category |
|---|E
F
G
H

## Slide 2
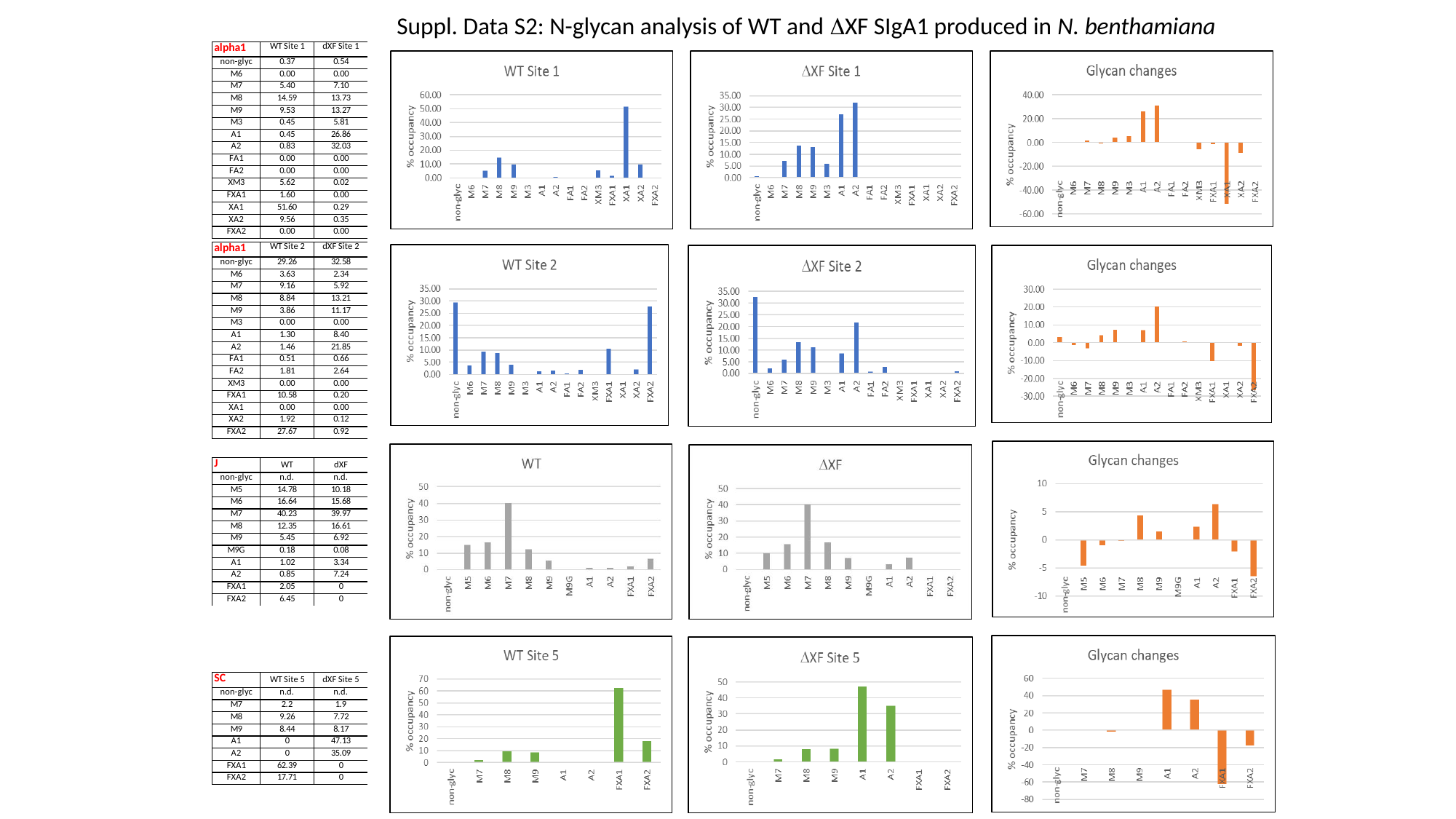

Suppl. Data S2: N-glycan analysis of WT and DXF SIgA1 produced in N. benthamiana
D

## Slide 3
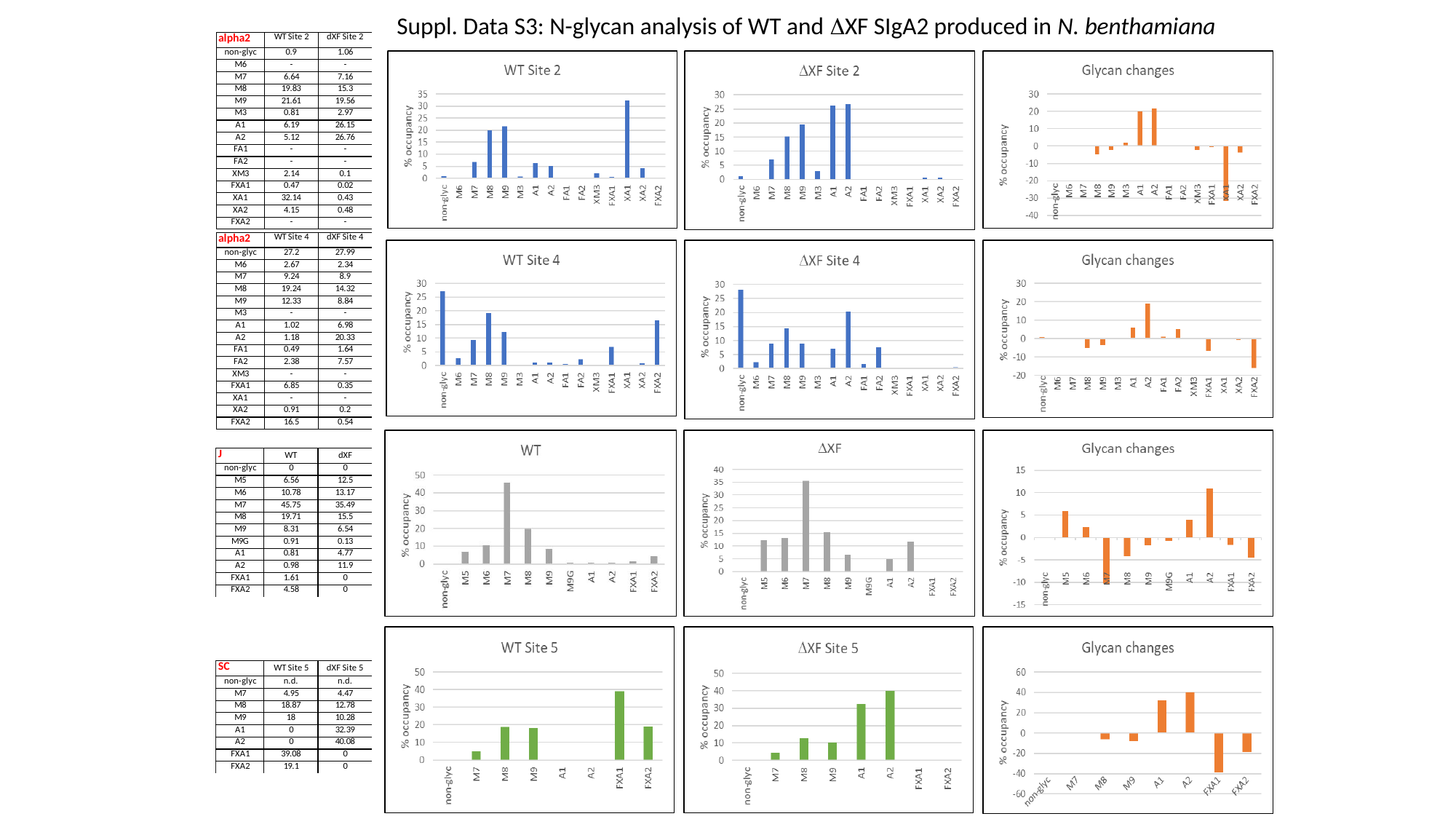

Suppl. Data S3: N-glycan analysis of WT and DXF SIgA2 produced in N. benthamiana

## Slide 4
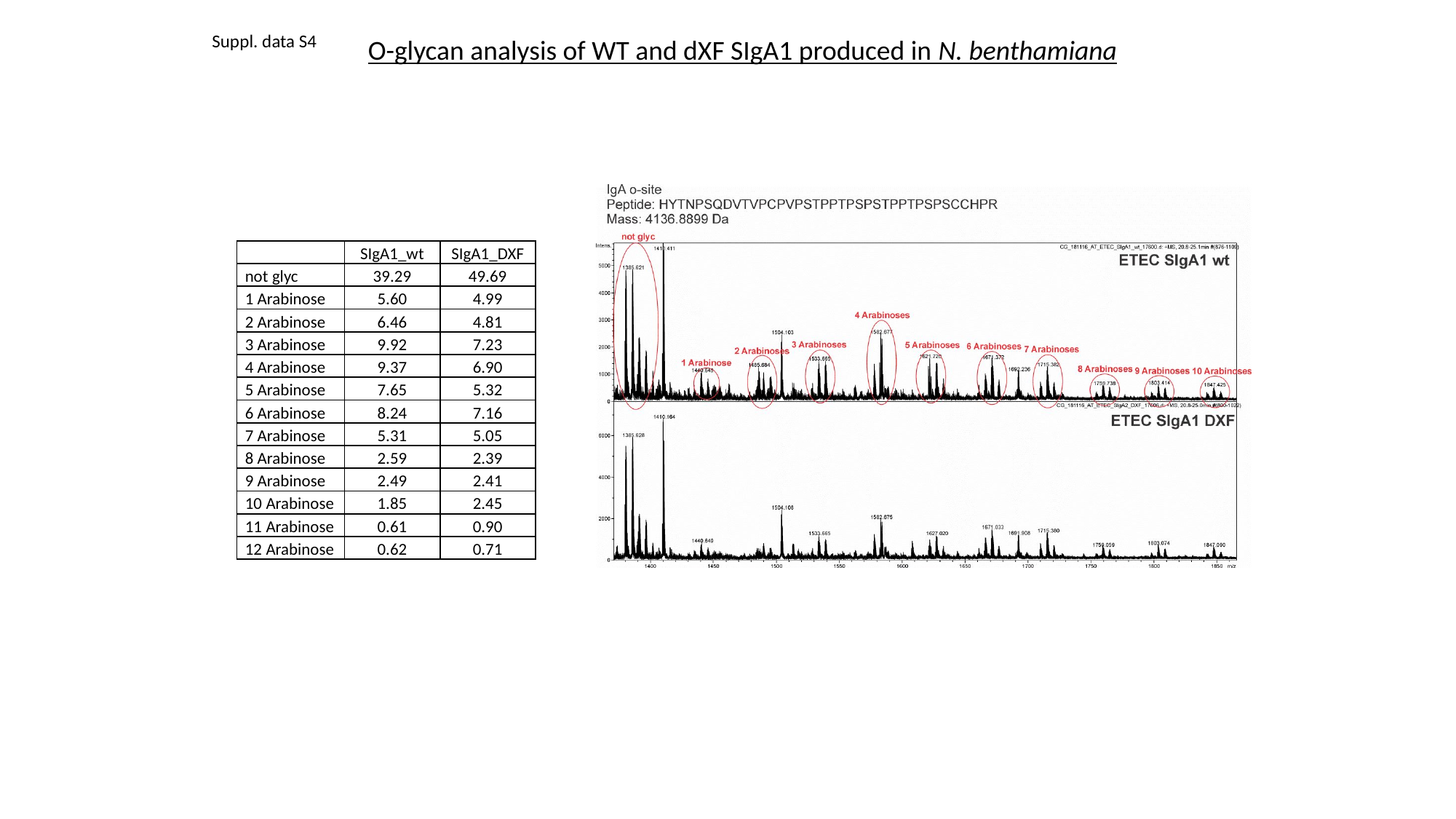

Suppl. data S4
O-glycan analysis of WT and dXF SIgA1 produced in N. benthamiana
| | SIgA1\_wt | SIgA1\_DXF |
| --- | --- | --- |
| not glyc | 39.29 | 49.69 |
| 1 Arabinose | 5.60 | 4.99 |
| 2 Arabinose | 6.46 | 4.81 |
| 3 Arabinose | 9.92 | 7.23 |
| 4 Arabinose | 9.37 | 6.90 |
| 5 Arabinose | 7.65 | 5.32 |
| 6 Arabinose | 8.24 | 7.16 |
| 7 Arabinose | 5.31 | 5.05 |
| 8 Arabinose | 2.59 | 2.39 |
| 9 Arabinose | 2.49 | 2.41 |
| 10 Arabinose | 1.85 | 2.45 |
| 11 Arabinose | 0.61 | 0.90 |
| 12 Arabinose | 0.62 | 0.71 |
