# Supplementary material for: Investigation of a monoclonal antibody against enterotoxigenic Escherichia coli, expressed as secretory IgA1 and IgA2 in plants
Source: Gut Microbes. 2021 Jan 13;13(1):1859813. doi: 10.1080/19490976.2020.1859813 (PMC7833773; doi:10.1080/19490976.2020.1859813)
Supplement: Supplemental Material [file KGMI_A_1859813_SM5311.docx]

**Supplemental figures**

**S1: Analytical size exclusion chromatography.**  Representative size exclusion chromatograms of sIgA1 (A-D) and sIgA2 (E-G) produced in CHO (B, F), wt Tobacco (C, G) and ΔXF Tobacco (D, H). Elution time of gel filtrations standards are indicated on the top of every graph.

**S2: N-glycan analysis of SIgA1 produced in WT and ΔXF *N. benthamiana:*** Glycans associated with N-glycosylation sites 1 and 2 of the alpha heavy chain, J chain and SC component site 5 are shown for tobacco produced SIgA1. Numbers represent percentage of total glycan composition. The Oxford glycan nomenclature was used for glycan abbreviations.

**S3: N-glycan analysis of SIgA2 produced in WT and ΔXF *N. benthamiana:*** Glycans associated with N-glycosylation sites 2 and 4 of the alpha1 heavy chain, J chain and SC component site 5 are shown for tobacco produced SIgA1. Numbers represent percentage of total glycan composition. The Oxford glycan nomenclature was used for glycan abbreviations.

**S4: O-glycan analysis of SIgA1 produced in WT and ΔXF *N. benthamiana:*** Relative proportions of glycoforms associated with IgA1 O-glycosites. The mass spectrometry spectrum is shown below. For quantification, the peak areas of EICs (Extracted Ion Chromatograms) of the first four isotopic peaks were summed, using the quantification software Quant Analysis (Bruker). The Oxford glycan nomenclature was used for glycan abbreviations.
